# Cancer genome landscape: a radiologist’s guide to cancer genome medicine with imaging correlates

**DOI:** 10.1186/s13244-019-0800-0

**Published:** 2019-11-28

**Authors:** Francesco Alessandrino, Daniel A. Smith, Sree Harsha Tirumani, Nikhil H. Ramaiya

**Affiliations:** 1 Department of Imaging, Dana Farber Cancer Institute, Harvard Medical School, 450 Brookline Avenue, Boston, MA 02215 USA; 2 Department of Radiology, Brigham and Women’s Hospital, Harvard Medical School, 75 Francis Street, Boston, MA 02115 USA; 3 Department of Radiology, UH Cleveland Medical Center, Case Western Reserve University, 11100 Euclid Ave, Cleveland, OH 44106 USA

**Keywords:** Cancer, Genetic heterogeneity, Mutation, Molecular targeted therapy, Signal transduction

## Abstract

The introduction of high throughput sequence analysis in the past decade and the decrease in sequencing costs has made available an enormous amount of genomic data. These data have shaped the landscape of cancer genome, which encompasses mutations determining tumorigenesis, the signaling pathways involved in cancer growth, the tumor heterogeneity, and its role in development of metastases. Tumors develop acquiring a series of driver mutations over time. Of the many mutated genes present in cancer, only few specific mutations are responsible for invasiveness and metastatic potential, which, in many cases, have characteristic imaging appearance. Ten signaling pathways, each with targetable components, have been identified as responsible for cancer growth. Blockage of any of these pathways form the basis for molecular targeted therapies, which are associated with specific pattern of response and toxicities. Tumor heterogeneity, responsible for the different mutation pattern of metastases and primary tumor, has been classified in intratumoral, intermetastatic, intrametastatic, and interpatient heterogeneity, each with specific imaging correlates. The purpose of this article is to introduce the key components of the landscapes of cancer genome and their imaging counterparts, describing the types of mutations associated with tumorigenesis, the pathways of cancer growth, the genetic heterogeneity involved in metastatic disease, as well as the current challenges and opportunities for cancer genomics research.

## Key points

- Landscapes of cancer genome and their imaging counterparts are introduced.
- The pathways of cancer growth targeted by targeted therapies are described.
- Imaging presentation of metastatic disease reflects the genetic heterogeneity of metastases.
- The current challenges and opportunities for cancer genomics research are described.

## Introduction

Cancer is a disease of the gene. Any change to genes that control cell growth and division may potentially lead to cancer. Nonetheless, of the many genetic mutations occurring in a cell, only a minimal part will cause cancer, whereas the wide majority will have no impact on cell survival. It is evident that the key to decipher cancer genesis is through identification of those genetic mutations occurring in cells that lead to cancer [1].

**Fig. 1:**
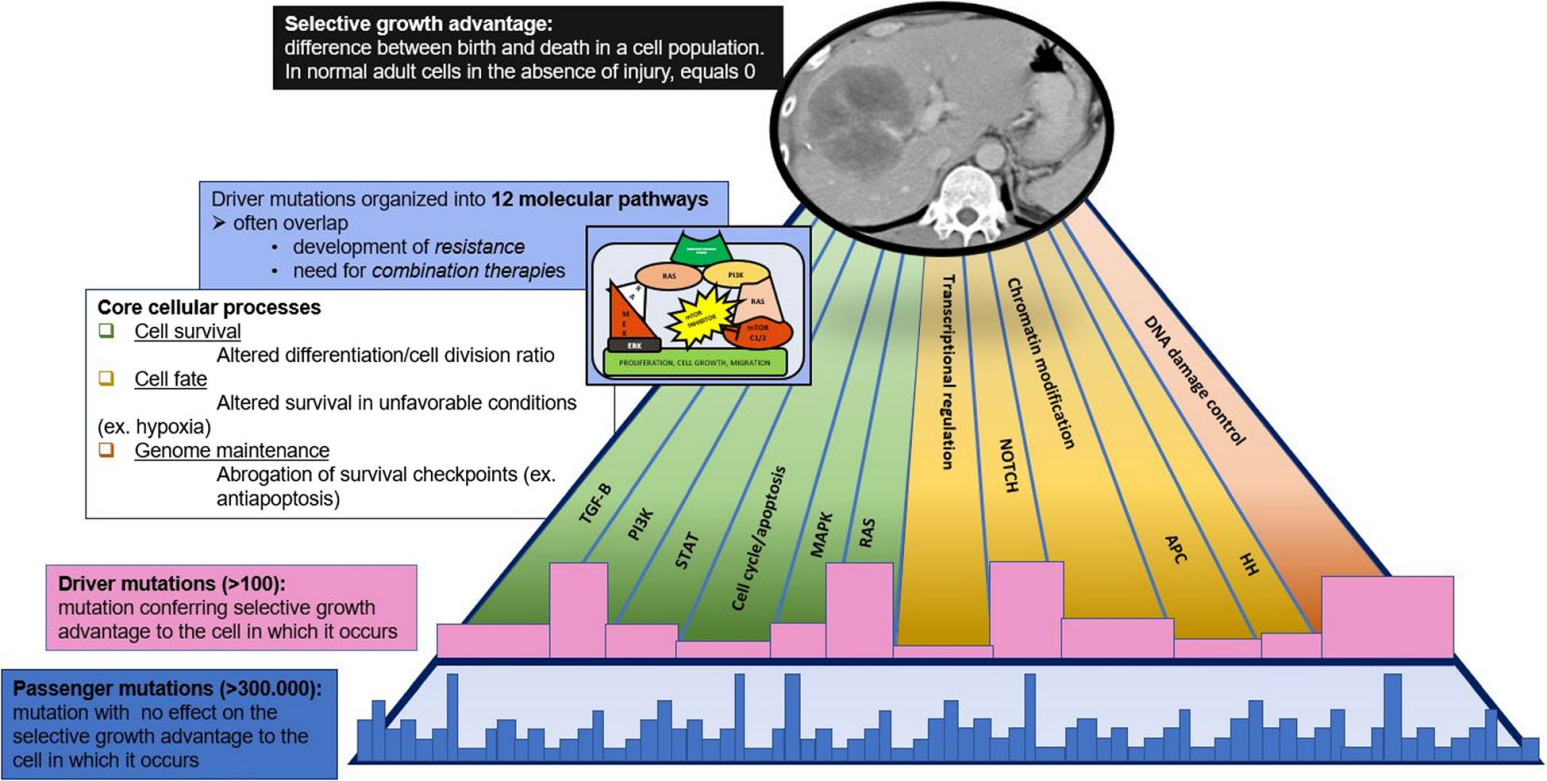
Cancer genome landscapes: structure and glossary

The introduction of high throughput sequence analysis in the past decade and the hundred-fold decrease in sequencing costs, from more than 100,000$ to 1000–2000$ for analysis of a single cancer genome case, has made whole genome cancer analysis possible [2]. This has made available an enormous amount of genomic data: a recent whole genome analysis of 33 cancer types identified 1,457,702 different mutations [3, 4]. Although vast amount of data can be easily obtained, the challenge for the oncologic scientific community is to understand the role of mutations in cancer genesis and survival and their clinical implications. An extraordinary effort was carried out in the past decades to reveal the common mutations occurring in human cancer, to decipher the role of these mutations in carcinogenesis, to define the molecular pathways of cancer development and growth, and to understand the cause and mechanism of tumor heterogeneity (Fig. 1) [4, 5]. These efforts helped to shape the cancer genome landscape, giving a comprehensive view of cancer growth and development.

Similarly, the high-throughput mining of image features from medical images allows to build correlations between qualitative or quantitative imaging findings and each component of the cancer genome landscape, from mutation driven carcinogenesis, to pathway-specific cancer growth and development of metastases [6, 7].

Understanding the mechanisms of cancer genesis and the landscapes of cancer genomics is crucial to anyone involved in oncologic patient care, including radiologists [8, 9]. In clinical practice, cancer-related imaging studies, from screening to diagnosis and disease staging, represent indeed a large segment of the imaging studies performed in most radiology departments. Radiologists, from the large academic centers to the small efficient private practices, are crucial for the clinical care of cancer patients, and should be familiar with the landscapes of cancer genome to understand the imaging phenotype of the disease, to interpret the response to treatment to oncologic drugs and the imaging presentation of response.

In this article, we will introduce the key components and the imaging counterparts of the landscapes of cancer genome, describing the types of mutations associated with tumorigenesis, the pathways of cancer growth, the genetic heterogeneity involved in metastatic disease, and the current challenges and opportunities for cancer genomics research. For every component, relevant imaging examples will be presented.

## Cancer genome mutations and tumorigenesis

The number of genes containing somatic mutations in tumor is highly variable, ranging from thousands of mutations in microsatellite colorectal cancer to less than ten in leukemia and pediatric tumors [5, 10]. The vast majority of these mutations are single-base substitutions, while the remaining 5% are base deletions or insertions [11–13]. Tumors develop acquiring a series of mutations over time, with a mechanism that has been widely studied in colorectal cancer: the adenoma-carcinoma sequence starts with a “gatekeeping” mutation which provides selective growth advantage to a normal cell over adjacent cells, most commonly occurring in the *APC* gene, then a second mutation occur allowing clonal expansion of the mutated cell and eventually other mutations occur, determining further growth of the clone and giving invasive characteristics to the mutated cell [14].

A representative example of these phenomenon which can be observed on imaging is histologic transformation of indolent lymphoma: follicular lymphoma, a common type of indolent non-Hodgkin lymphoma, can transform into the aggressive diffuse large b cell lymphomas (DLBCL) by means of the stepwise acquisition of a set of mutations, most notably involving *TP53* [15, 16]. Transformation to DLBCL can be suspected on cross-sectional imaging when lymph node enlargement, disproportionate to the rest of the nodal involvement is noted; when lymph nodes show areas of decreased density on CT or increased T2 hyperintensity on MRI, reflecting areas of necrosis, or when new extranodal lesions are noted [17, 18]. On FDG positron emission tomography (PET)/CT, transformed lymph nodes show higher FDG uptake with increased SUVmax when compared to other non-transformed nodes in the same patient, or when new FDG-avid lesions are noted in various organs, with increased SUVmax compared to the rest of the disease [18, 19]. Prompt identification of transformation is crucial, as treatment and prognosis differ among the two.

### Cancer genome mutations

To understand the complexity of the mutations involved in tumorigenesis, these have been classified into passenger mutations, occurring in the “preneoplastic” phase with no effect on neoplastic process, and driver mutations, responsible for invasiveness and metastatic potential [5, 20].

Mutations conferring selective growth advantage and ultimately responsible for tumorigenesis are termed driver mutations. On average, an adult cancer needs 1–8 driver gene mutations to occur. Given their critical role in the process of oncogenesis, it is not surprising that unique driver mutations have been associated with specific imaging characteristics and response patterns on diagnostic imaging. For example, research in the field of radiogenomics have sought to differentiate imaging features of different molecular subtypes of non-small cell lung cancer (NSCLC) based on specific driver mutations, including anaplastic lymphoma kinase (*ALK*) or epithelial growth factor receptor (*EGFR*). EGFR-mutated NSCLC, for instance, has been associated with specific CT features such as higher rates of pleural retraction, homogeneous enhancement, smaller size, oval shape, and fewer calcifications [21–23].

Identifying which genes contain driver mutations is challenging, and current data are derived from studies analyzing the frequency of mutated genes in a given cancer type. Among the mutated genes in a cancer, the ones more commonly mutated are more likely to contain driver mutations, whereas the less frequently mutated genes, yet numerically more present in cancers, are less likely to contain driver mutations: from sequencing of 3284 tumors, only 125 driver genes for 294,881 mutations were identified [24, 25]. Of these, 54 were oncogenes and 71 were tumor suppressor genes. In addition, genes expressed aberrantly in tumors, yet not frequently mutated are involved in tumorigenesis, and are termed epi-genes. These are altered through changes in DNA methylation or chromatin modification [5].

### Dark matter

Cancer development and progression cannot be explained only in terms of driver and passenger mutations: in many cancers, only one or two driver mutated genes are identified. This contradicts the somatic evolution model, in which multiple sequential mutations acquired over decades are needed for a cancer to arise [14]. This apparent contradiction can be only partially resolved considering the technical and conceptual limitations of whole genome sequencing: many driver mutations cannot be identified with current sequencing techniques, and the vast majority of cancer genome studies focused at identifying mutations at exon levels, ignoring intergenic or intronic mutations [5]. In addition, epi-genes are extremely difficult to sequence, yet are often responsible for carcinogenesis. Nonetheless, the “dark matter” responsible for cancer development has not been fully understood.

## Signaling pathways and cancer growth

Driver genes activate cancer growth through ten signaling pathways, which act on three cellular processes: cell survival, cell fate, and genome maintenance (Fig. 1) [5, 26]. Almost all of the currently available conventional and novel targeted therapies act on one or the other pathway. These pathways often interact and overlap with each other. These signaling pathways form the foundation of tumorigenesis and serve as a framework for modern targeted cancer therapies. Understanding these signaling pathways is especially critical for radiologists, as these pathways have key imaging correlates (Table 1) [29]. Integrating knowledge of these signaling pathways into modern diagnostic image interpretation is a critical skill in the age of modern genomic-based oncology.

**Table 1:** Core cellular processes, signaling pathways, genomic mutations in cancers, and identified imaging correlates

Core cellular process	Signaling pathway	Mutation	Cancer	Imaging findings
Cell fate	Notch	*NOTCH1* [27]	ALL, HNSCC, CLL	No specific imaging findings
	HH	*PTCH1* [28]	BCC	No specific imaging findings
	APC	*APC*	Colon	No specific imaging findings for sporadic mutation. In germline mutation, imaging findings associated with familial adenomatous polyposis or Turcot syndrome (extracolonic polyps, osteomas, dental anomalies, sebaceous cysts, hepatoblastomas, glioblastoma or medulloblastomas, papillary thyroid cancer, desmoid, and soft-tissue tumors) [29]
	Transcriptional regulation	*ER*/*PR*	Breast	Later development and higher frequency of bone metastases on scintigraphy and lower frequency of brain metastases on brain MRI, compared to ER/PR^−^ breast cancer [29, 30]. ER^+^ breast cancers: smaller with irregular borders and low ADC values on breast MRI; associated with low accuracy of MRI in predicting residual tumor extent after neoadjuvant systemic therapy, when compared to triple negative or HER^+^ breast cancers [31, 32]
		*AR*	Prostate	ADC values in tumor increase after therapy [33]. In castrate-resistant prostate cancer, increasing sclerosis of bone metastases correlates with decreased SUVmax [34] [35].
	Chromatin modification	*H2A*, *H2B*, *H3*, *H4* [36]	CTCL	No specific imaging findings
Cell survival	RAS^a^	*EGFR*	NSCLC	More commonly associated with air bronchograms, pleural retraction, small lesion size, and absence of fibrosis, than EGFR-wild type NSCLC [37, 38]. In patients with exon 21 mutation, groundglass opacity morphology and volume are significantly higher than in patients with exon 19 mutation or EGFR wild-type NSCLC [39]. More commonly spiculated morphology and more commonly present with bone and lung metastases compared to its ALK-mutated counterpart [40]
			Colon	Initial 20% decrease in tumor size after 8 weeks of cetuximab correlates with better overall response and longer progression-free survival [41]
		*HER2*	Breast	Tend to be multicentric and multifocal with nodal involvement. More commonly associated with liver metastases than HR^+^ breast cancer. Increased risk of central nervous system relapses particularly if already treated with trastuzumab [42]
			NSCLC	Disseminated lung nodules and tumor excavation patterns observed with high frequency [43]
		*KIT*	GIST	GIST with KIT exon 11 mutations are commonly gastric in origin, shows better tumor response on follow-up imaging and lower rates of disease recurrence following treatment with imatinib compared to GIST with exon 9 mutations, which more commonly originates from small bowel [24, 25, 44, 45]; a sizeable proportion of these patients will develop resistance to treatment within six months due to secondary mutations in exon 13 or 17 [52]
		*KRAS*	NSCLC	Round lesion shape, nodules in non-tumor lobes more common than KRAS-wild type NSCLC [37]
			Colon	KRAS mutation associated with lung and brain metastases, and recurrence in lungs [46, 47]
		*VEGF*-*A*	HCC, RCC, hypervascular tumors	Hypervascularity at contrast-enhanced CT/MR/US [29]
	MAPK^a^	*BRAF* MEK	Melanoma	Response to BRAF/MEK inhibitors combination therapy
		*BRAF*	Colon	Decreased response to EGFR inhibitors compared to wild-type [35]
	STAT^a^	*JAK2*	Polycythemia vera	No specific imaging findings
	PI3K	*PIK3CA, AKT1*, *mTOR*	CLL, RCC, breast, neuroendocrine	No specific imaging findings
	Cell cycle/apoptosis	*CDK4*/*6*	Breast, ovarian	No specific imaging findings
		*BCL*-*2*	CLL	No specific imaging findings
	TGF-β	*TGFB1*	Breast, metastatic cancers	No specific imaging findings
Genome maintenance	DNA damage control	*BRCA1*, *BRCA2* *TGFB1*	Breast	Predilection for posterior breast and prepectoral region. Fibroadenoma-like benign morphologic features such as oval/round shape and smooth margins, or non-mass like enhancement on MRI for BRCA1-mutated breast cancer [48]. Low prevalence of calcifications on mammogram in BRCA1-mutated breast cancer, more common with BRCA2 mutation [48]
			Ovarian	Commonly shows peritoneal disease, peritoneal spread of disease in the gastrohepatic ligament, supradiaphragmatic lymphadenopathy and mesenteric involvement on CT [49]

*CLL* chronic lymphocytic leukemia, *NSCLC* non-small cell lung cancer, *GIST* gastrointestinal stromal tumor, *HCC* hepatocellular carcinoma, *RCC* renal cell carcinoma, *ALL* acute lymphoblastic leukemia, *HNSCC* head and neck squamous cell carcinoma, *BCC* basal cell carcinoma, *CTCL* cutaneous T cell lymphoma, *AR* androgen receptor

^a^Part of RTK-RAS pathway

### Cell fate

Pathways acting on cell fate alter the ratio between differentiating cells, which cannot undergo division, and dividing cells, shifting the ratio toward the latter, conferring selective growth advantage to the tumor [5, 50].

Pathways that function through this process include gene regulation by steroid hormones, which can be targeted by hormonal therapies, and chromatin modifications, which can be targeted by drugs inhibiting histone deacetylases [50–52].

The first pathway is exemplified by estrogen and progesterone receptor-positive (ER/PR^+^) breast cancer. These types of breast cancer show later development and higher frequency of bone metastases on scintigraphy and lower frequency of brain metastases on brain MRI, compared to their hormone receptor negative (HR^−^) counterparts [29, 30]. In addition, ER^+^ breast cancers tend to be smaller with irregular borders and low ADC values on breast MRI and are associated with low accuracy of MRI in predicting residual tumor extent after neoadjuvant systemic therapy, when compared to triple-negative or HER^+^ breast cancers [31, 32].

**Fig. 2:**
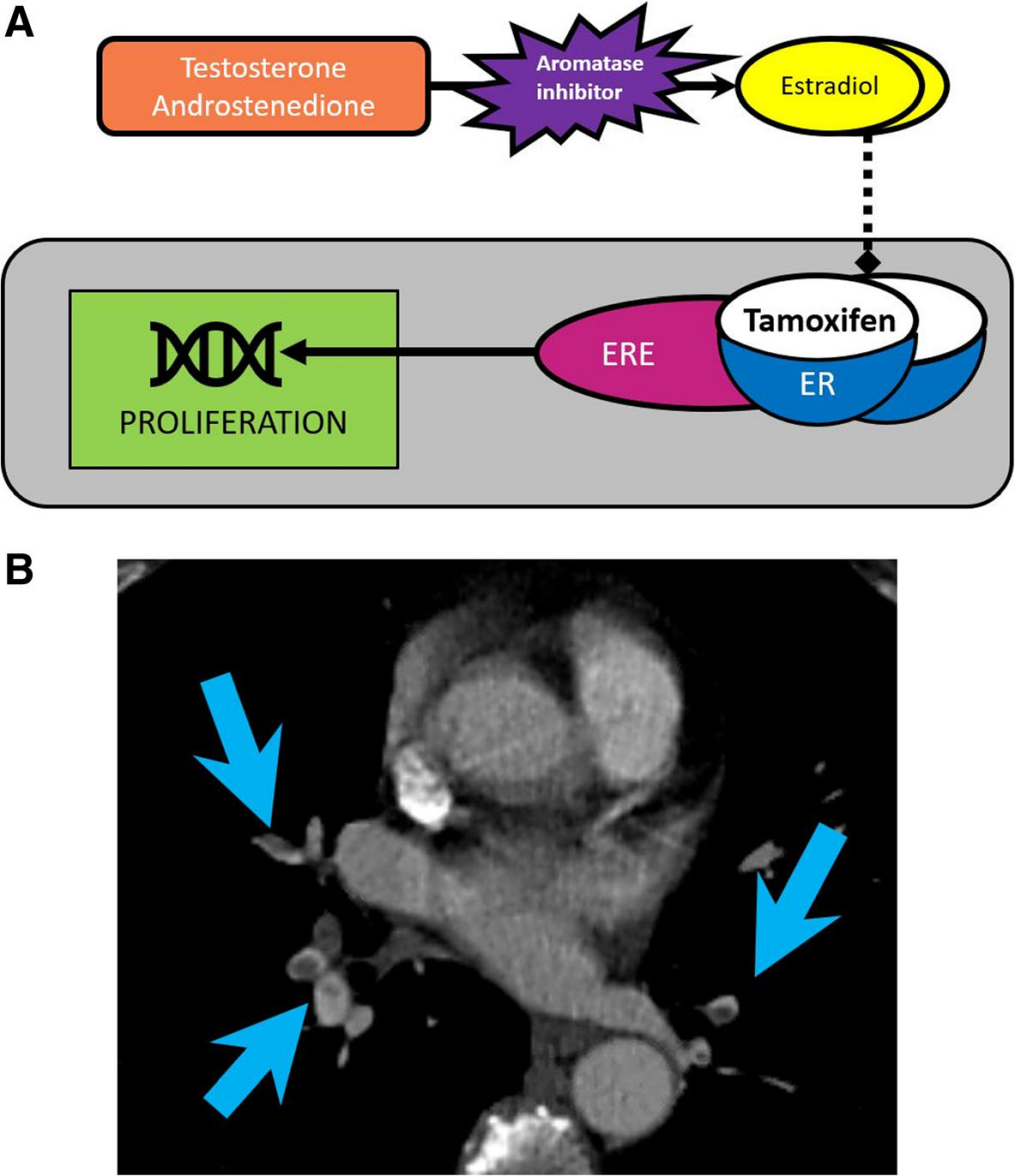
Transcriptional regulation pathway by steroid hormones. **a** Image showing the role of aromatase inhibitors and tamoxifen in blocking steroid synthesis. Estradiol binds to estrogen receptor (ER), leading to dimerization and binding to estrogen response elements (ERE) activating estrogen-responsive genes leading to proliferation. Tamoxifen competes with estradiol for ER binding aromatase inhibitors decreasing the synthesis of estrogens from their precursors. **b** Contrast-enhanced CT images of the chest and abdomen in a 72-year-old woman with ER^+^ invasive lobular breast cancer treated with letrozole (aromatase inhibitor) and tamoxifen. CT of the chest shows multiple filling defects in the bilateral segmental pulmonary arteries (arrows), compatible with pulmonary embolism, an adverse event associated with tamoxifen

This pathway is targeted by tamoxifen, selective estrogen receptor modulator, and anastrozole or letrozole, two FDA-approved aromatase inhibitors to treat HR^+^ breast cancer (Fig. 2) [53, 54].

The chromatin modification pathway is targeted by histone deacetylases inhibitors, which include vorinostat, approved by the FDA for cutaneous T cell lymphoma treatment. Histone deacetylase enzymes switch cells from quiescent to replicative status and in addition, increase cell death by apoptotic and non-apoptotic mechanisms [55]. Histone deacetylases inhibitors keep cells in quiescent status, avoiding initiation of cell replication [56].

### Cell survival

**Fig. 3:**
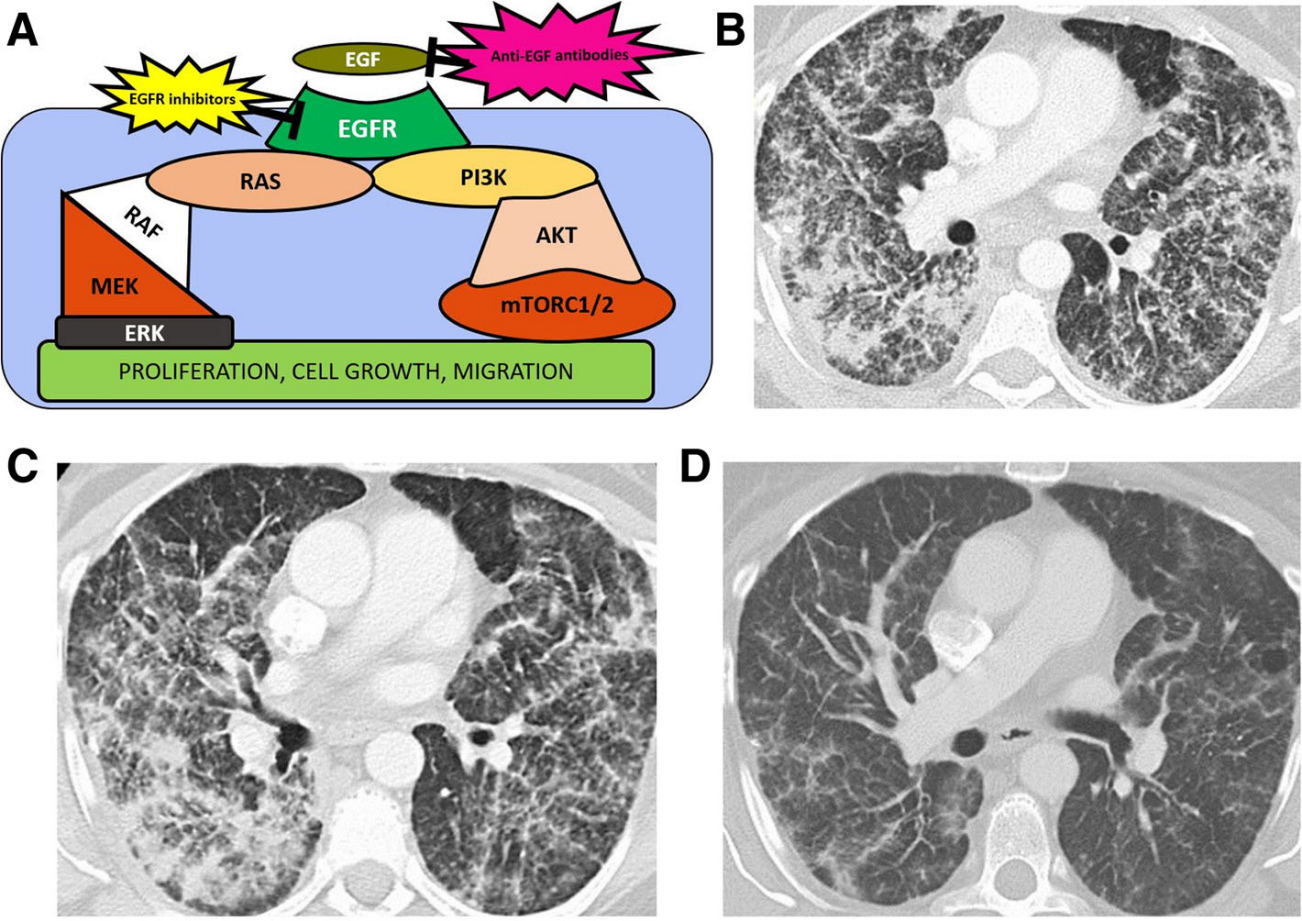
RTK-RAS pathway. **a** Image showing the RAS-RTK pathway, activated by epithelial growth factor (EGF) and its receptor (EGFR). RAS and EGF/EGFR activate PI3K-RAS-mTORC and RAS-RAF-MEK pathways, determining cell proliferation and cell growth. The pathway is blocked by EGF antibodies, such as cetuximab or EGFR inhibitors such as erlotinib or afatinib (**b**–**d**) contrast-enhanced CT images of the chest 64-year-old woman with multifocal adenocarcinoma of the lung with mutation of the EGFR exon 21. Patient was initially treated with erlotinib and follow up CT (**b**) at 2 months after treatment was started shows mild improvement of the lung consolidative opacities compared to baseline CT of the chest (**a**). Patient developed erlotinib associated shortness of breath and rash, and was switched to another EGFR inhibitor, afatinib. **c** CT of the chest performed 6 months after afatinib was started, shows significant resolution of the consolidations and of the interlobular thickening

**Fig. 4:**
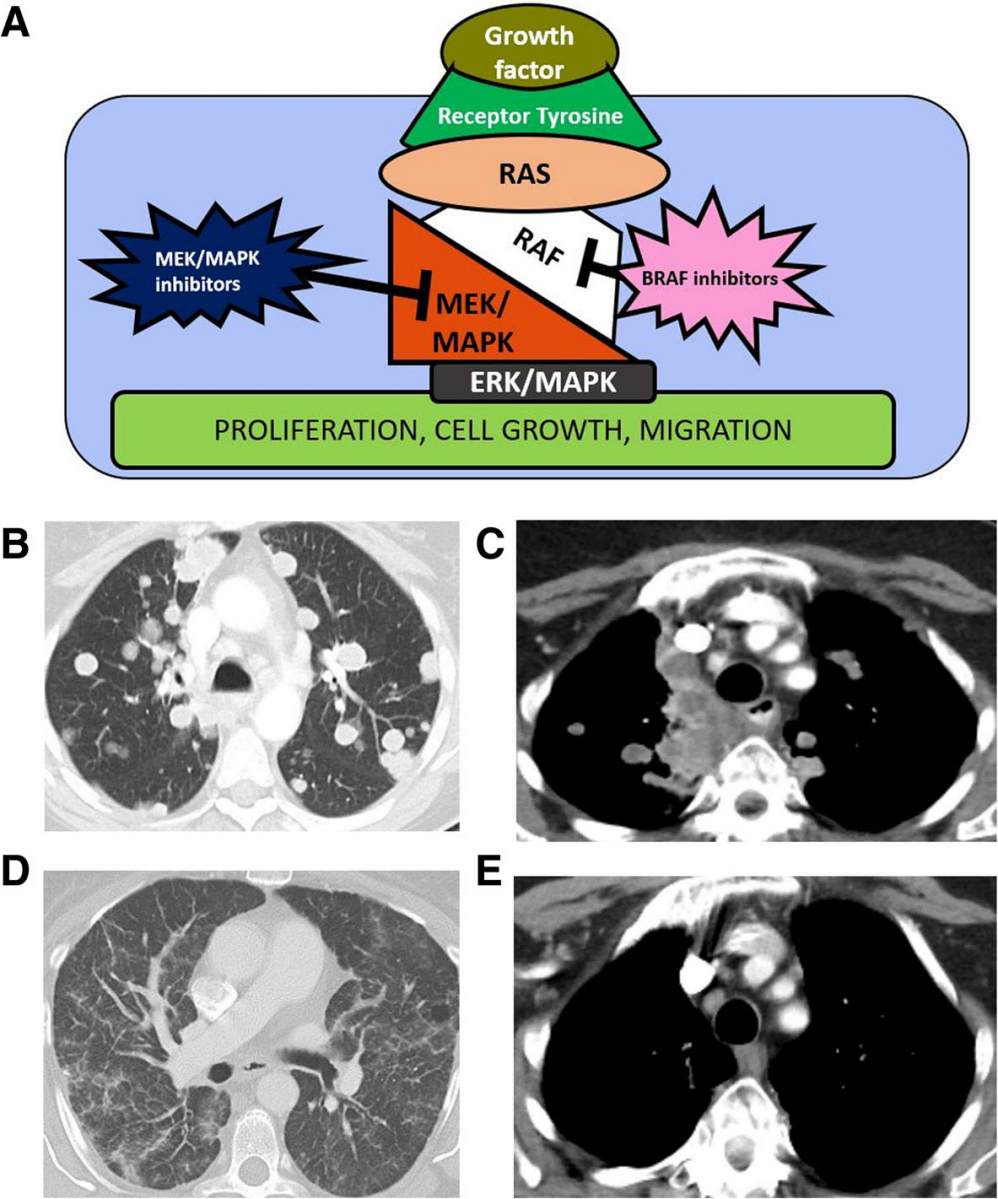
RTK-RAS pathway. **a** Image showing the activation of the MAPK transcription factor by binding of growth factor to a transmembrane receptor tyrosine kinase. The resulting signaling cascade culminates with translocation of ERK/MAPK to the nucleus, where ERK activates transcription factors that ultimately result in cell growth. BRAF inhibitors, such as dabrafenib, block RAF signal and MEK/MAPK inhibitors, such as trametinib, block the MEK/MAPK signal. **a**–**d** Contrast-enhanced CT images of the chest of a 52-year-old woman with BRAF V600 mutant melanoma, progressed on ipilimumab, treated with dabrafenib and trametinib. Baseline (**a**, **b**) and follow-up CT (**c**, **d**) performed two months after treatment was started, shows almost complete resolution of multiple lung nodules and mediastinal masses

**Fig. 5:**
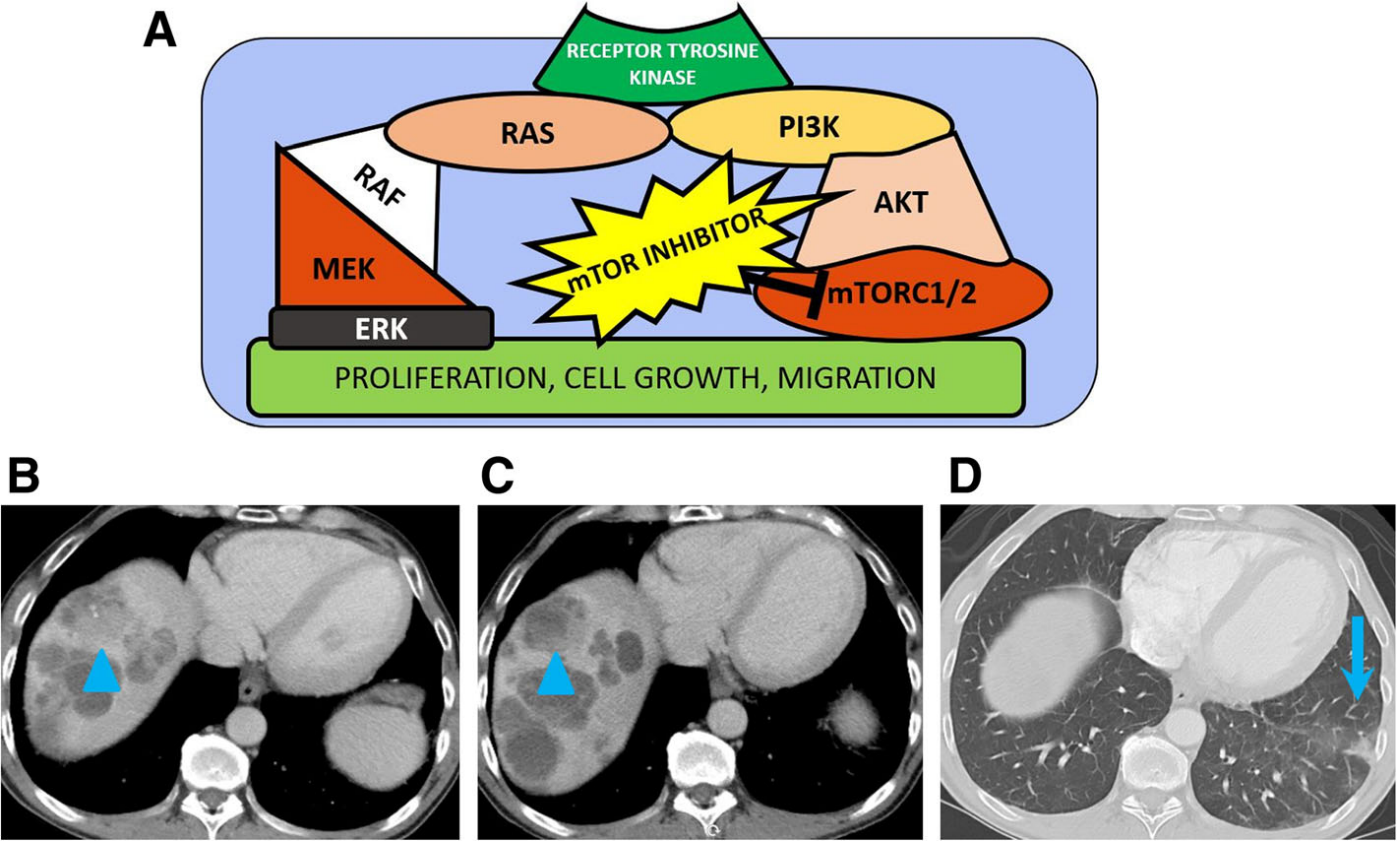
PI3K pathway. **a** Image showing the PI3K-ATK-mTORC pathway and the blockage of the effector mTORC1/2 by the mTOR inhibitors, such as everolimus. **b**–**d** Baseline and restaging contrast-enhanced CT images of a 60-year-old man with metastatic neuroendocrine tumor on everolimus show decreased enhancing component of a liver metastasis (arrowheads), representing response to treatment. Patient developed drug induced pneumonitis while on treatment (arrow) (**d**)

Cancer cells which can proliferate in unfavorable conditions, such as hypoxia or low glucose levels, will have a selective growth advantage compared to healthy cells. Mutations in the various pathways encoding for receptors for growth factors or for proteins involved in downstream cell growth pathways allow survival in unfavorable conditions. Identified pathways targeting cell survival are the receptor tyrosine kinase (RTK)/RAS, PI3K/AKT/mechanistic target of rapamycin (mTor), pathways regulating cycle cell and apoptosis, and TGF-beta pathway (Figs. 3, 4, and 5).

The RAS/RAF/MEK pathway, the MAPK pathway, and the JAK/STAT pathways share common tyrosine kinase receptors and transcription factors and are currently thought to be part of the RTK-RAS pathway, which is one of the most well-studied signaling pathways with critical role in cell cycle progression and growth. In this pathway, activation of epidermal growth factor receptor (EGFR) by its associated growth factor EGF and other RTKs leads to downstream activation of RAS GTPases and RAF kinases such as BRAF. Subsequent activation of additional kinase proteins such as MEK and MAPK follows in this signal pathway. These pathways ultimately lead to translocation of ERK/MAPK to the nucleus with subsequent transcription factor phosphorylation that regulates cell growth (Figs. 3 and 4) [57].

Mutations in the genes along the RTK/RAS pathway can occur at different levels, from the growth factors receptors to the downstream effectors [5, 9, 26]. Imaging correlates of mutations along this pathway include the EGFR-mutant NSCLC. When compared to EGFR wild-type NSCLC, this is more commonly associated with air bronchograms, pleural retraction, small lesion size, and absence of fibrosis [37, 38]. In patients with exon 21 mutation, groundglass opacity morphology and volume are significantly higher than in patients with exon 19 mutation or EGFR wild-type NSCLC [39]. In addition, EGFR-mutated NSCLC shows more commonly spiculated morphology and more commonly present with bone and lung metastases compared to its ALK-mutated counterpart [40]. The clinically important RTK-RAS signaling pathway serves as the basis for modern EGFR inhibitors, now a critical pillar of modern oncologic therapeutic management across numerous malignancies. In normal cells, when EGFR binds to an extracellular growth factor, various signaling pathways are activated, including the PI3K/AKT/mTor, JAK/STAT, and the RAS/RAF/MAPK pathways. EGFR inhibitors such as erlotinib and gefitinib are commonly employed for the targeted treatment of NSCLC testing positive for EGFR mutations, which are associated with pulmonary toxicity (Fig. 3) [58].

EGFR-mutated or EGFR-overexpressing colorectal cancer is associated with higher rates of radiological tumor response to cetuximab, an anti-EGF antibody, and response to treatment may be predicted by early decrease in tumor size on follow-up imaging acquired by 8 weeks after cetuximab initiation has also been associated with improved long-term survival [41, 59]. Cetuximab has also been associated with development of interstitial pneumonitis with CT findings of new bilateral patchy areas of ground glass attenuation [60, 61].

HER2-mutated breast cancers tend to be multicentric and multifocal with nodal involvement, are more commonly associated with liver metastases than the HR^+^ breast cancer, and have increased risk of central nervous system relapses particularly if already treated with trastuzumab, a monoclonal antibody binding to the extracellular domain of HER2 [42]. HER2-mutated NSCLC frequently present with disseminated lung nodules and tumor excavation patterns [43].

Another RTK that has been extensively studied is KIT, a type III receptor kinase composed of an extracellular ligand, a transmembrane region, and an intracellular domain with a juxtamembrane region and tyrosine kinase domains [8]. After ligand binding, KIT causes phosphorylation and subsequent downstream activation of the RAS/RAF/MAPK, JAK/STAT, and PI3K/AKT/mTor signaling pathways [62]. Mutations in *KIT* gene are present in 85% of gastrointestinal stromal tumors (GISTs), commonly at exon 11, which confers increased sensitivity to imatinib, a KIT tyrosine kinase inhibitor, and at exon 9, which confers increased sensitivity to sunitinib, a vascular endothelial growth factor receptor inhibitor which also targets KIT [63]. Specific KIT mutational variants have been associated with different initial imaging presentations, response patterns, and recurrence patterns. For example, GIST patients with exon 11 mutations tend to experience better tumor response on follow-up imaging and lower rates of disease recurrence following treatment with imatinib compared to patients with exon 9 mutations, which portends a more aggressive course [24, 25, 44]. Despite the improved prognosis associated with exon 11 mutations, a sizeable proportion of these patients will develop resistance to treatment within 6 months due to secondary mutations in exon 13 or 17 [44]. For patients harboring these secondary mutations, radiologists should be aware that these patients are at high risk of developing recurrence and progressive disease on subsequent imaging while on treatment with imatinib.

Patients with mutations in BRAF (most commonly BRAFV600) and MEK, two effectors in the RTK/RAS pathway, can be treated with BRAF and MEK inhibitors [5]. Available BRAF inhibitors utilized for the treatment of advanced BRAF-mutant melanoma include vemurafenib and dabrafenib. Tumor response to BRAF inhibitors is often rapid (Fig. 4). Nonetheless, development of acquired resistance ultimately occurs in the majority of cases with evidence of progressive disease on follow-up imaging. Combination therapy with BRAF and MEK inhibitors has been shown to reduce the rate of acquired resistance and lead to improved response rates in BRAFV600-mutant advanced melanoma [64, 65]. Combination BRAF/MEK inhibitor therapy is thus now the standard targeted therapy treatment, with available BRAF/MEK inhibitor combinations including vemurafenib/cobimetinib, dabrafenib/trametinib, and encorafenib/binimetinib.

The JAK tyrosine kinase and the STAT transcriptor factors play a key role in regulating cellular proliferation of hematopoietic precursor cells. Mutations in *JAK2* leading to constitutive activation of the JAK-STAT pathway are common in polycythemia vera [66]. Ruxolitinib is an FDA-approved JAK1/JAK2 inhibitor for treatment of myelofibrosis and polycythemia vera [67].

Constitutive activation of PI3K/AKT/mTor pathway by a mutated receptor tyrosine kinase leads to the chronic production of growth factor ligands, which send signals to normal cells to supply growth factors, increasing receptor proteins on the cancer cell surface to make them more sensitive to growth factor ligand [68]. In addition, mutations along the PI3K/AKT/mTor pathway increase migration, proliferation, and motility. Many types of cancer show mutations along the PI3K/AKT/mTor pathway including neuroendocrine tumors, renal cell carcinoma, breast cancer, perivascular epithelioid cell tumors, and gastrointestinal stromal tumors [68].

The PI3K/AKT/mTor pathway is targeted by mTor inhibitors including everolimus, currently approved by the FDA for various cancers, including locally advanced, unresectable, or metastatic neuroendocrine tumors of pancreatic origin, and advanced renal cell carcinoma after failure of treatment with sunitinib or sorafenib (Fig. 5) [69].

Other mechanisms which confer selective growth advantage to cancer cells involve mutations of genes regulating cycle cell and apoptosis.

When growth factors stimulate quiescent cells to enter the cell cycle, D-type cyclins associate with CDK4/6 to promote cell cycle progression through G1 phase [70]. Palbociclib and ribociclib inhibit CDK4/6-Cyclin D action in promoting cell cycle progression and are FDA approved for HR^+^ HER2 negative advanced and/or metastatic breast cancer in combination with an aromatase inhibitor in postmenopausal women.

Apoptosis, or programmed cell death, acts as a defense against cancer growth [71]. Various proteins, including BCL-2, inhibit apoptosis by binding to and suppressing proapoptotic proteins [71]. Resistance to apoptosis occurs in chronic lymphocytic leukemia, which is associated with elevated BCL-2 protein expression [8]. Venetoclax, a BH3-mimetic FDA-approved drug for 17p-deleted refractory chronic lymphocytic leukemia, antagonizes BCL-2 and induces apoptosis [72].

TGF-β promotes tumorigenesis via a variety of mechanisms, making this pathway an emerging pathway for potential targeting in anticancer treatments. Various drugs targeting this pathway are currently under investigation for treatment of different cancers, although none has been granted FDA approval [73].

### Genome maintenance

Any cell is exposed to toxic substances present in the microenvironment in which they reside. Replicative checkpoints prevent damaged cells to progress into the cell cycle or force the damaged cell to undergo apoptosis. Cells with mutations in genes regulating these checkpoints will have selective growth advantage compared to nonmutated cells [5]. Genes whose mutations acts on these checkpoints include *TP53*, *ATM*, and *BRCA* which are observed in breast and hereditary pancreatic cancers [74].

*BRCA1* and BRCA*2* are tumor suppressor genes which activate specific DNA repair processes in cases of damaged DNA. BRCA1-mutated breast cancer show low prevalence of calcifications on mammogram, more common with BRCA2 mutation. In addition, BRCA1-mutated breast cancer shows predilection for posterior breast and prepectoral region and show fibroadenoma-like benign morphologic features such as oval/round shape and smooth margins, or non-mass-like enhancement on MRI [48]. BRCA-mutated high grade serous ovarian cancer commonly shows peritoneal disease, peritoneal spread of disease in the gastrohepatic ligament, supradiaphragmatic lymphadenopathy, and mesenteric involvement on CT [49].

**Fig. 6:**
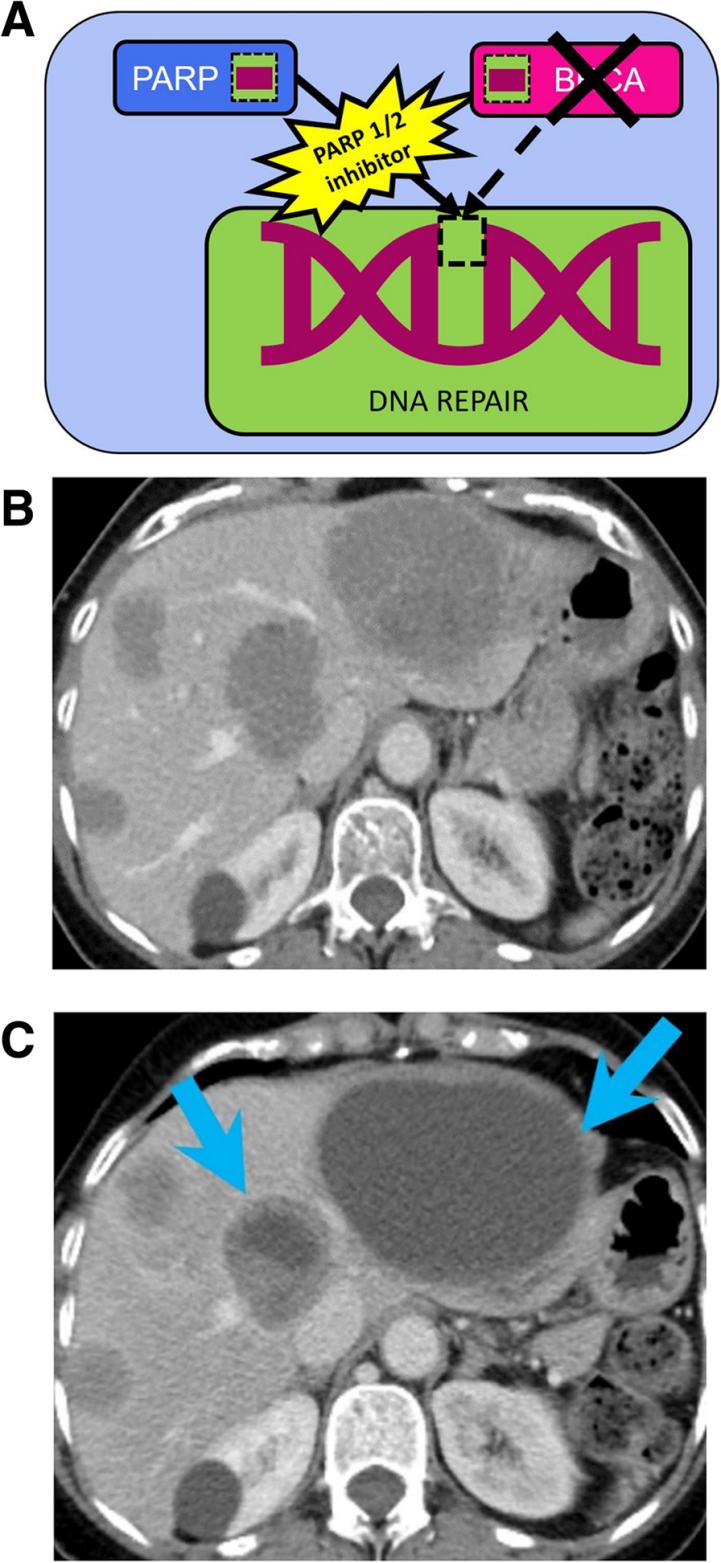
Genome maintenance and DNA damage control pathway. **a** Image showing the role of poly (ADP-ribose) polymerases (PARP) in DNA repair. PARP1 and PARP2 repair DNA single- or double-DNA strand breaks. In patients with *BRCA* loss-of-function mutations, PARP 1/2 inhibitors, such as niraparib, are particularly effective as BRCA contribute to DNA repair. **b**, **c** CT of the abdomen in 65-year-old woman with platinum resistant ovarian cancer on treatment with niraparib. **a** Baseline contrast-enhanced CT image before starting niraparib shows multiple hypodense large liver metastases. **b** Follow-up contrast-enhanced CT image performed two months after starting the treatment shows decreased enhancing component of the various lesions (arrows in **c**), with minimal interval increase in size in some of the lesions, representing atypical response to treatment

Poly (ADP-ribose) polymerases (PARP) 1/2 inhibitors are two targeted agents particularly effective in patients with mutated *BRCA*. In patients with *BRCA* loss-of-function mutations, PARP1 and -2 repair DNA single- or double-DNA strand breaks (Fig. 6) [75, 76]. PARP inhibition can cause genetic errors with double-strand breaks that ultimately lead to cell death [8, 77].

Various PARP inhibitors are currently used in clinical practice, including olaparib, niraparib, and talazoparib which have been granted FDA approval for treatment of ovarian or BRCA-mutated breast cancer. Resistance to PARP inhibitors represent a major barrier to the survival of patients with BRCA1- and BRCA2-mutated cancers and is thought to be related to various mechanisms, including mutations in *BRCA*, restoring its DNA repair function (Fig. 6) [77, 78]. Patients with ovarian carcinoma who develop secondary mutations in *BRCA1/2* are not only likely to develop resistance and progressive disease on follow-up imaging when treated with platinum chemotherapy but also may similarly develop resistance to PARP inhibitors. Radiologists should thus be aware that these secondary mutations increase the likelihood that subsequent follow-up imaging will demonstrate increased tumor burden [78, 79].

## Development of metastases and cancer heterogeneity

**Fig. 7:**
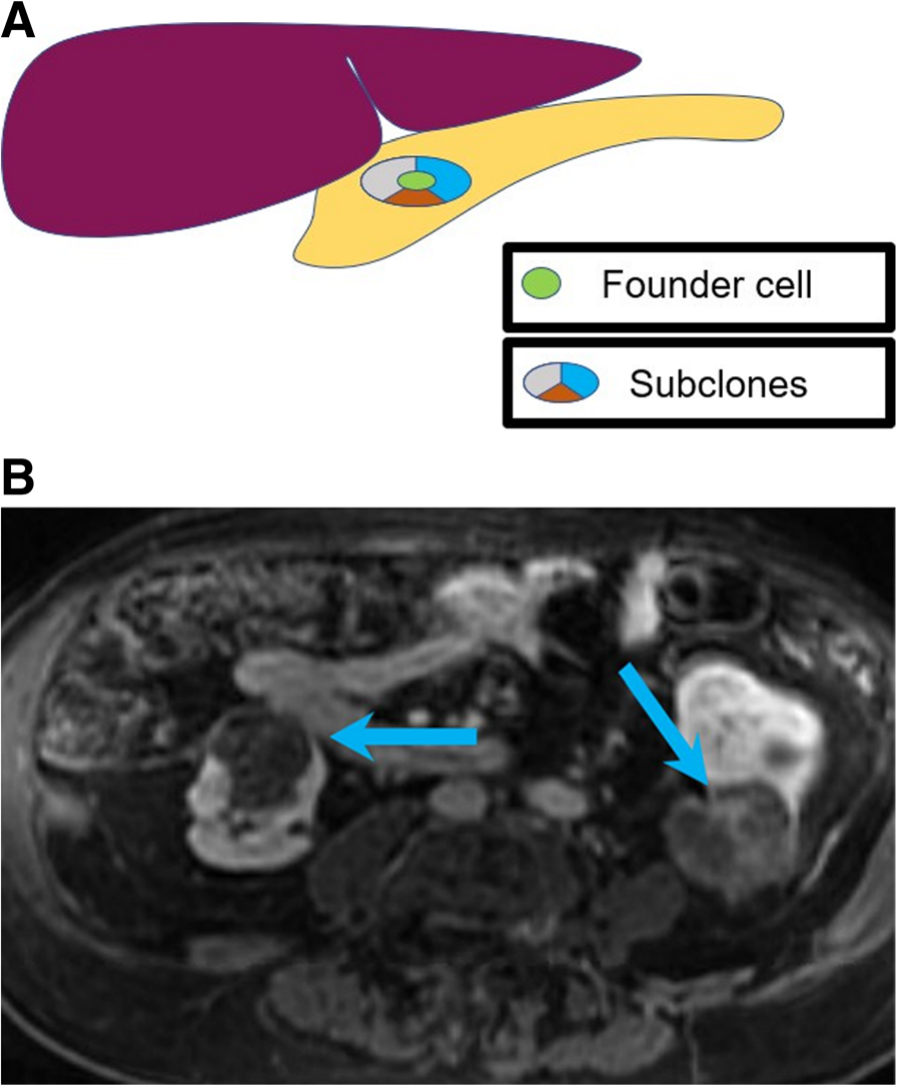
Intratumoral heterogeneity. **a** Image exemplifying the heterogeneity within the subclonal cells of a pancreatic tumor, all derived from a founder cell. **b** Fat-saturated T1-weighted postcontrast MRI image acquired during arterial phase showing heterogeneously appearing renal cell carcinomas (blue arrows). Mutations can be missed at tissue sampling obtained from biopsies, given the mutational heterogeneity of renal cell carcinoma

Genetic alterations responsible for the development of metastasis remain to be identified. Nonetheless, a few phenomenon related to metastases development were observed in genome cancer studies: metastatic potential can be present early in tumorigenesis, years before metastasis occur and mutations in metastases are highly heterogeneous, sometimes differing from the mutations present in the primary tumor [80]. Tumor heterogeneity, in turn, is related to the presence of subclonal mutations, which are present in only some of the mutated cells of the primary tumor and are observed in metastases. So far, four types of tumor heterogeneity have been described:

- Intratumoral heterogeneity, defined as the heterogeneity occurring within the cells of one tumor, which reflects the difference in the subclonal cells of the same tumor all derived from a founder cell [81]. Intratumoral heterogeneity is frequently observed in oncologic imaging (Fig. 7). As a representative example, large (> 5 cm) GISTs are more commonly heterogeneous in appearance and have higher metastasizing potential than their smaller, low risk counterparts [82].

This phenomenon poses the basis for radiologic-genomic correlation, which is made possible through the extraction and reproducible quantification of morphologic and quantitative metrics observed on medical imaging, a process termed radiomics [83]. Data on tumor heterogeneity imaging can be extracted from imaging studies with various methods, including analysis of simple qualitative descriptors, such as size, shape, margins; non-spatial quantification of parameter distributions with histogram-based analysis; quantification of spatial complexity such as texture analysis (a mathematical method to evaluate the gray-level intensity and position of the pixels within an image); or quantitative assessment of spatial distribution of parameters [84–88].

These data can be acquired from different imaging techniques, including dynamic contrast-enhanced CT, US, or MRI; perfusion CT; diffusion-weighted imaging; magnetic resonance spectroscopy; arterial spin-labeling; blood oxygenation level-dependent MR imaging; elastography; and PET imaging [86].

**Fig. 8:**
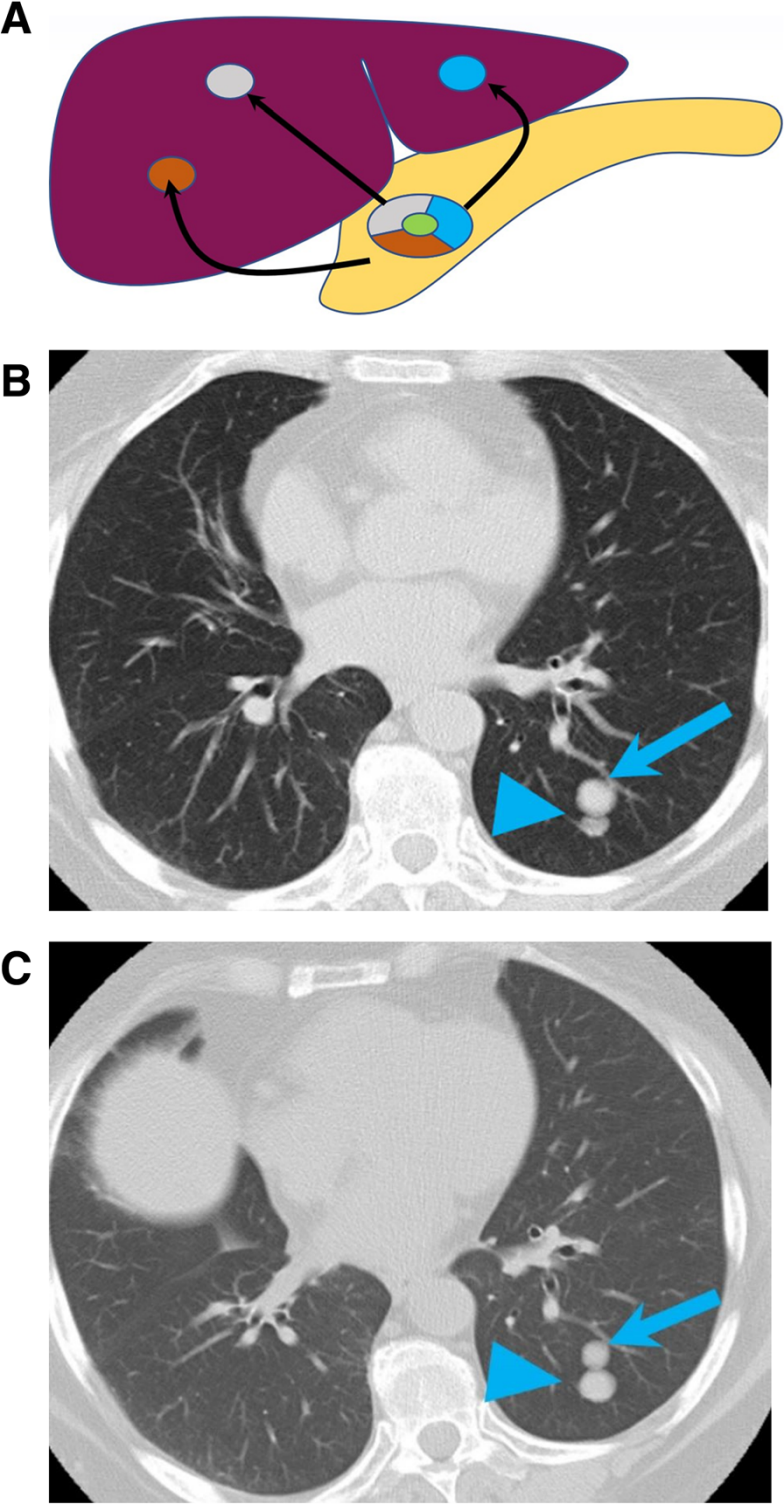
Intermetastatic heterogeneity. **a** Image showing the heterogeneity within different liver metastases, each one arising from a different subclone in a primary pancreatic tumor. **b** Baseline and (**c**) follow-up axial CT images of the chest of a 77-year-old woman with leiomyosarcoma on pazopanib shows increase in size of a nodule (blue arrowhead), and interval decrease in size of an adjacent nodule (blue arrow), representing different response to the vascular endothelial growth factor inhibitor pazopanib

**Fig. 9:**
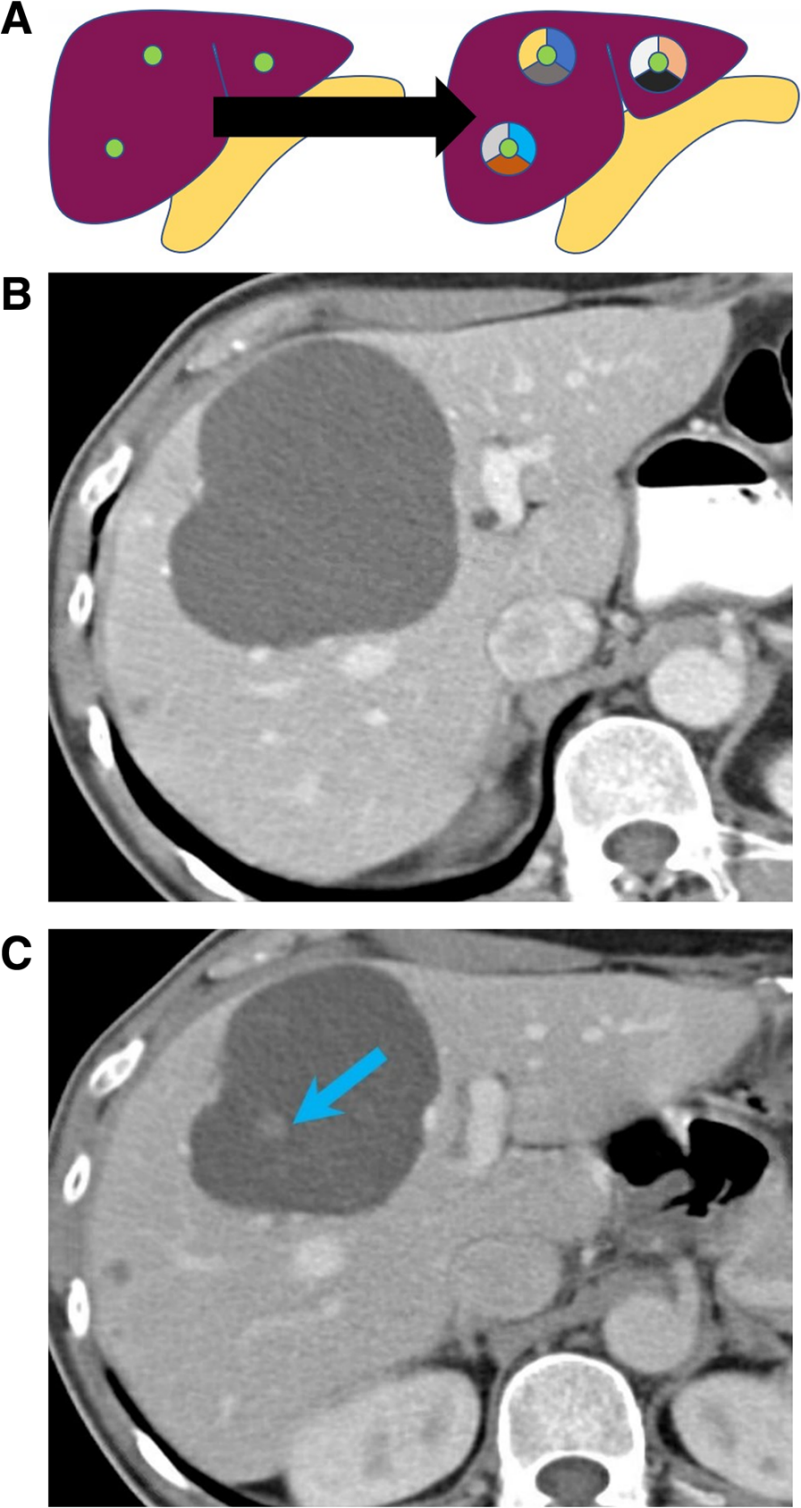
Intrametastatic heterogeneity. **a** Image exemplifying the development of heterogeneity among the cells the liver metastases as the metastases grow (black arrow). Axial CT images acquired during portal venous phase (**b**, **c**) in a 77-year-old woman with gastrointestinal stromal tumor acquired during treatment with imatinib. The hypodense metastasis in the liver shows a new intralesional soft tissue nodule at follow up scan (arrow) (**c**), suspicious for recurrence

**Fig. 10:**
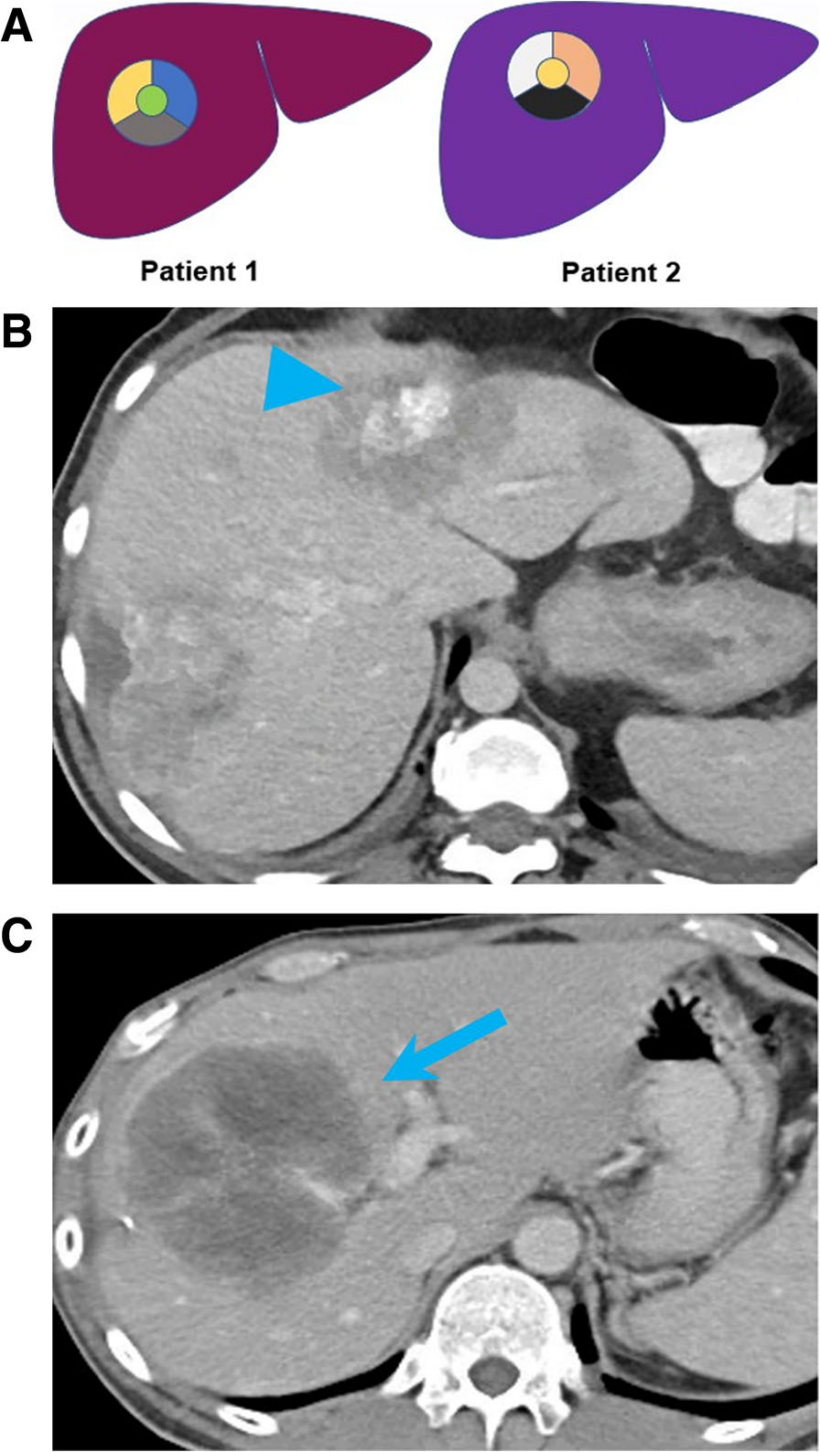
Interpatient heterogeneity. **a** Image showing mutational heterogeneity among tumors of different patients, where different mutations are depicted in different colors. **b**, **c** Axial CT images acquired during portal venous phase in two different patients with pathological diagnosis of metastatic adenocarcinoma of the colon. One patient shows partially calcified metastases (arrowhead) (**b**); one patient shows hypodense heterogeneously enhancing masses (**c**)

(b) Intermetastatic heterogeneity: heterogeneity within the different metastases, each one arising from a different subclone [89]. This could explain the mixed response to treatments in metastatic cancers, with lesions responding to molecular targeted treatments and other developing resistance. Intermetastatic heterogeneity is a phenomenon commonly observed on restaging studies of patients with metastatic diseases treated with molecular targeted therapies. This occurs when metastatic lesions responding to therapy, and progressing metastatic lesions, coexist at the same timepoint. This phenomenon has been observed in more than one-third of patients with non-small cell lung cancer treated with EGFR tyrosine kinase inhibitors and can be often misinterpreted as progression on conventional imaging response criteria [90]. On imaging studies, this is exemplified when metastatic lesions increasing in size or enhancement and metastatic lesions decreasing in size or enhancement coexist in the same patient (Fig. 8). The heterogeneous response to treatment in different metastases, reflects the heterogeneous biological characteristics of different lesions within the same patient.
(c) Intrametastatic heterogeneity: heterogeneity among the cells of each metastasis develops as the metastases grow [5]. This phenomenon is represented by recurrence after response in a metastatic lesion. On imaging, intermetastatic heterogeneity can be observed in patients with metastatic GIST treated with imatinib. The nodule-within-a-mass is an imaging pattern described in patients with GIST in which a new enhancing solid nodule develops into a treated hypodense lesion, representing the clonal selection and growth of clusters of mutant cells with new genomic mutations, commonly occurring in KIT, determining resistance to imatinib (Fig. 9) [91]. Identification of this pattern, representing resistance to imatinib, on restaging CT or MR of patients with metastatic GIST is crucial, allowing prompt modification of therapy.
(d) Interpatient heterogeneity: heterogeneity among same tumor types of different patients. This occurs when two patients with the same tumor types shows different imaging and histologic presentation or different treatment response when treated with the same compound (Fig. 10) [92]. An example of interpatient heterogeneity comes from GISTs: the biologic behavior of GIST tends to be determined by the mutational status, and this is ultimately reflected on imaging. GISTs are associated with various mutations, including mutations of *KIT*, which can occur at exon 9 or exon 11, *SDH*, or *PDGFRA*, and the type of mutation predicts the imaging characteristics of the disease, the response to treatment, and the prognosis [45]. While GIST with *KIT* mutations at exon 11 are often gastric in origin and respond dramatically to the tyrosine kinase inhibitor imatinib, GISTs with KIT exon 9 mutations are often small bowel in origin and have aggressive course [45]. SDH-deficient GISTs tend to be multifocal, frequently metastasize to nodes, have an indolent course in spite of metastasis, and are commonly resistant to imatinib [93].

## Challenges

The limitations of cancer genome-based medicine reside in the current therapeutic approach of modern oncology. First, virtually all clinically approved drugs that target the products of genetically altered genes in oncology are directed against kinases. Although kinases are relatively easy to identify and target, this limits the possible targets of molecular therapies, since most of the known oncogenes have complex biological activities which goes beyond the enzymatic activity of kinases. In addition, many driver mutated genes encode tumor suppressor which are difficult to target by clinically available drugs. Finally, a pressing issue of molecular targeted therapies is represented by development of drug resistance, which are responsible of short-term remissions with most of current therapies. The use of combination therapies, allowing inhibition of downstream feedbacks and multiple pathways, delays the occurrence of resistance, nonetheless needs more than one targetable genetic alteration to work.

## Opportunities

**Fig. 11:**
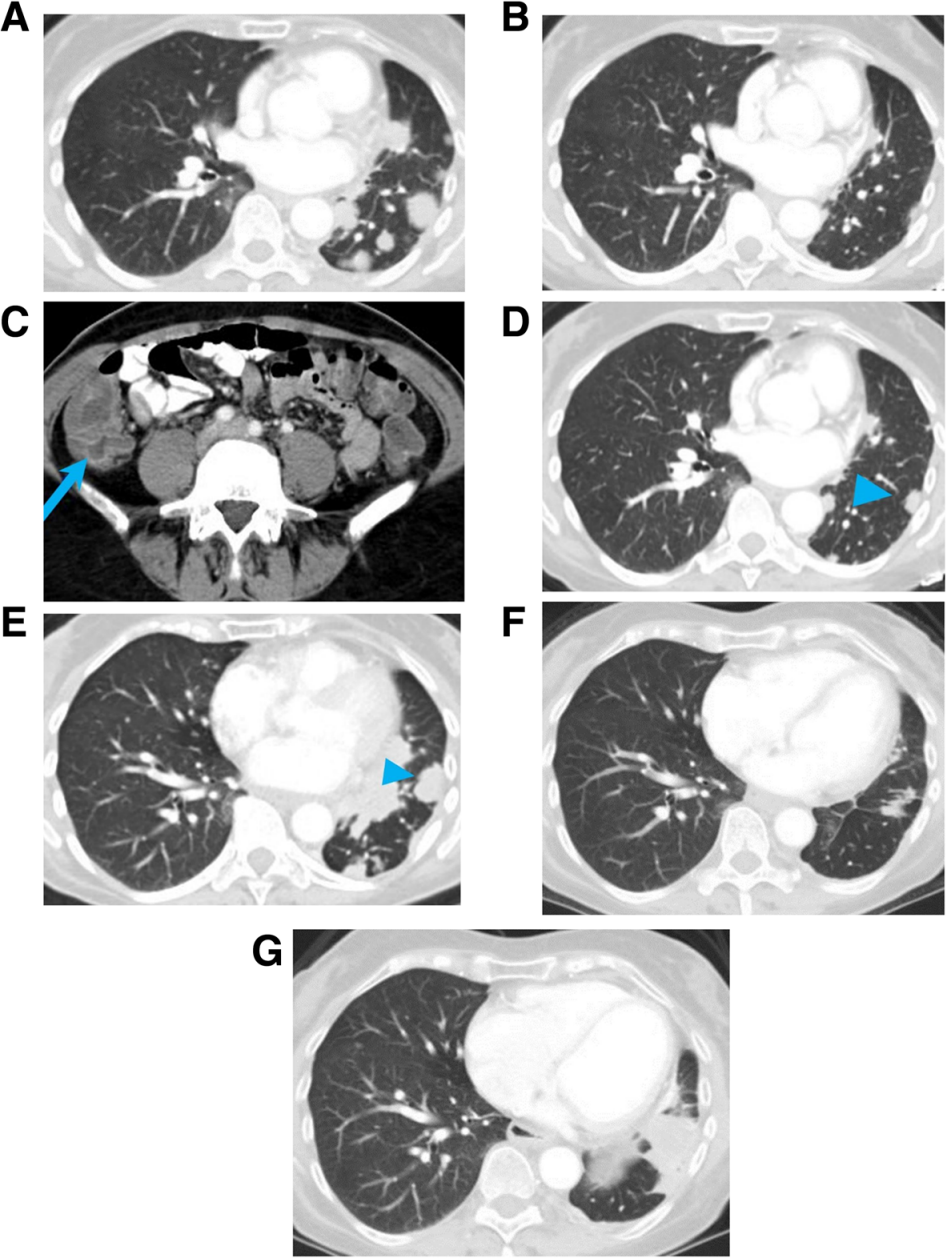
A 68-year-old nonsmoker woman with non-small cell lung cancer, with *EGFR* exon 19 deletion. Baseline CT of the chest (**a**) shows multiple lung nodules, which shrunk after 3 months of treatment with the EGFR inhibitor erlotinib (**b**). While on treatment, patient developed diarrhea, and CT of the abdomen acquired during portal venous phase showed fluid filled large bowel, consistent with drug induced colitis (arrow) (**c**). After 4 years of treatment, CT of the chest (**d**) showed a new left lower lobe nodule, which markedly increased in size in 3 months (arrowhead) (**e**). Biopsy of the nodule showed acquired T90M mutation, which confers resistance to erlotinib. Patient was switched to osimertinib, with initial improvement of the lung nodules on follow-up chest CT (**f**). Tumor burden remained stable for 2 years, until a chest CT scan showed increased right lower lobe nodule (**g**). Patient was then switched to pemetrexed

Genome-wide sequencing of various cancers has shed many lights on cancer development and progression. This knowledge can be used in many different ways, and in some cases, has already modified the therapeutic approach in clinical oncology. In many cases, expression of mutated gene in a cancer is tested before starting any therapy: the mutation of EFGR should be tested in all patients with NSCLC prior to start any systemic therapy and the presence of T790M resistance mutation, targetable by osimertinib, should be tested in patients with disease progression after first-line therapy with an EGFR inhibitor (Fig. 11).

Another possible, more futuristic therapeutic approach would be to modify dose of molecular targeted drug according to the levels of the drug metabolizing enzymes.

Finally, a potentially successful therapeutic approach would be integrating the knowledge derived from genome-wide sequencing of cancers with immunotherapy. This can be possible given that the proteins encoded by mutated genes acts as tumor-specific antigens and can be targeted by immune checkpoint inhibitors when presented by human leukocyte antigen (HLA) protein.

## Conclusion

The whole-genome sequencing of cancer has reshaped modern oncology. For radiologists, developing an understanding of the genomic basis of tumorigenesis and modern oncologic therapies provides useful insight into understanding initial presentation, response, and recurrence of cancer on diagnostic imaging. Although understanding cancer genomics seems a daunting task, having a finalistic view on cancer simplifies the cancer genome landscape: the final event of the many mutations in the cancer genome is to grant the cancer cell selective growth advantage, which depends on the activation of a relatively small number of cellular signaling pathways. Blockage of any of these pathways by molecular targeted therapies represents the current approach of oncologic therapy and allows understanding the occurrence of drug resistance and addressing therapeutic failure.

## Data Availability

Not applicable.

## Abbreviations

ALK: Anaplastic lymphoma kinase
DLBCL: Diffuse large b cell lymphoma
EGFR: Epithelial growth factor receptor
ER: Estrogen-receptor
GIST: Gastrointestinal stromal tumor
HR: Hormone receptor
mTOR: mechanistic target of rapamycin
NSCLC: Non-small cell lung cancer
PET: Positron emission tomography
PR: Progesterone receptor
RTK: Receptor tyrosine kinase
